# Development of Methanol Sensor Based on Sol-Gel Drop-Coating Co_3_O_4_·CdO·ZnO Nanoparticles Modified Gold-Coated µ-Chip by Electro-Oxidation Process

**DOI:** 10.3390/gels7040235

**Published:** 2021-11-26

**Authors:** Mohammed M. Rahman, Jahir Ahmed, Abdullah M. Asiri, Sulaiman Y.M. Alfaifi, Hadi. M. Marwani

**Affiliations:** 1 Department of Chemistry, Faculty of Science, King Abdulaziz University, P.O. Box 80203, Jeddah 21589, Saudi Arabia; amasirikau@gmail.com (A.M.A.); symalfaifi@gmail.com (S.Y.M.A.); hmmarwani@gmail.com (H.M.M.); 2 Center of Excellence for Advanced Materials Research (CEAMR), King Abdulaziz University, P.O. Box 80203, Jeddah 21589, Saudi Arabia; 3 Promising Centre for Sensors and Electronic Devices (PCSED), Advanced Materials and Nano-Research Centre, Najran University, P.O. Box 1988, Najran 11001, Saudi Arabia; jahmedsust@gmail.com

**Keywords:** methanol sensor, Co_3_O_4_·CdO·ZnO nanoparticles, tiny micro-chip, real sample analysis, electrochemical method

## Abstract

Herein, novel Co_3_O_4_·CdO·ZnO-based tri-metallic oxide nanoparticles (CCZ) were synthesized by a simple solution method in basic phase. We have used Fourier Transform Infrared Spectroscopy (FTIR), X-ray Diffraction (XRD), X-ray photoelectron spectroscopy (XPS), Field Emission Scanning Electron Microscope (FESEM), Dynamic Light Scattering (DLS), Tunneling Electron Microscopy (TEM), and Energy-Dispersive Spectroscopy (EDS) techniques to characterize the CCZ nanoparticles. XRD, TEM, DLS, and FESEM investigations have confirmed the tri-metallic nanoparticles’ structure, while XPS and EDS analyses have shown the elemental compositions of the CCZ nanoparticles. Later, a Au/μ-Chip was modified with the CCZ nanoparticles using a conducting binder, PEDOT: PSS (poly(3,4-ethylenedioxythiophene) polystyrene sulfonate) in a sol-gel system, and dried completely in air. Then, the CCZ/Au/μ-Chip sensor was used to detect methanol (MeOH) in phosphate buffer solution (PBS). Outstanding sensing performance was achieved for the CCZ/Au/μ-Chip sensor, such as excellent sensitivity (1.3842 µAµM^−1^cm^−2^), a wide linear dynamic range of 1.0 nM–2.0 mM (R^2^ = 0.9992), an ultra-low detection limit (32.8 ± 0.1 pM at S/N = 3), a fast response time (~11 s), and excellent reproducibility and repeatability. This CCZ/Au/μ-Chip sensor was further applied with appropriate quantification results in real environmental sample analyses.

## 1. Introduction

Methanol (MeOH) has attracted extensive interest for years because of its multipurpose utility in numerous fields [1,2]. It is a vital raw material in the production of glycol, olefins, formaldehyde, pesticides, etc., and is also noteworthy for use in direct methanol fuel cells [3]. However, the excessive use of MeOH can affect the environment, and even cause death, particularly in alcoholic beverages [4,5,6]. Therefore, it is essential to develop efficient techniques to detect MeOH accurately [7]. To date, many analytical techniques have been published for detecting MeOH, such as spectrophotometry, chromatography, electrochemistry, and colorimetry [8,9]. In comparison to these techniques, which require costly and sizeable equipment, the electrochemical technique is handy and less expensive, and also shows better selectivity and higher sensitivity in a quick response time [10].

To improve the sensing performance of electrochemical methanol sensors, various nanomaterials have been studied and applied [11,12]. Among them, Pt-group metals have been extensively investigated due to their excellent catalytic ability in relation to methanol [13]. However, Pt catalysts are easily poisoned by CO-like intermediates during reactions [14]. Moreover, the electro-oxidation of methanol on the Pt surface is also suggested to be a complex reaction involving a six-electron transfer along with a high activation energy [15]. To address the problem, Pt-based nanocomposites have been introduced to enhance the poison-tolerance capacity [16].

Lately, the electrochemical detection of hazardous chemicals by chemically modified electrodes (CMEs) has become vital due to their quick response, cheap method, handy nature, and high sensitivity, especially for in situ detection [17,18]. Developing an active material with better electro-catalytic activity and superior conductivity is the key feature of CMEs. Recently, the modification of an electrode by nanomaterials, such as transition metal oxides, various types of nanocomposites have become interesting research topics. Scientists have explored thin films consisting of composites of various mixed metal oxides to detect pollutants. Of these metal oxides, ZnO containing ternary metal oxides is an interesting material for sensing, since it gives a suitable environment for doping elements as a host, because of its high band-gap, low phonon frequency, and good thermal and chemical stability [19,20,21]. Hence, we have selected ZnO as the active methanol-sensing material. The incorporation of transition metals may affect the structural and optical properties of the materials. Therefore, we have simultaneously doped an n-type CdO and a p-type Co_3_O_4_ to the n-type ZnO to achieve a synergistic effect in the tri-metallic oxide [22,23,24]. The existence of electron–hole pairs in the active material has expedited a promising nano-environment for electrochemical oxidation [25,26,27,28]. So far, several phosphors have been reported on via the doping of copper and lanthanide combinations, but no such report is available for Co_3_O_4_·CdO·ZnO nanoparticles.

Herein, we have reported on the synthesis and systematic characterization of CCZ. Additionally, a Au-coated μ-Chip was fabricated by CCZ using the PEDOT:PSS in developing a MeOH sensor. A simple I-V method at ambient conditions was used in this study, since it is handy, low-cost, and requires less solvent, making it green. To the best of our knowledge, this will be the first report on a MeOH sensor based on its oxidation using CCZ as the active material.

## 2. Results and Discussion

### 2.1. Characterization of the CCZ Nanoparticles

**Figure 1:**
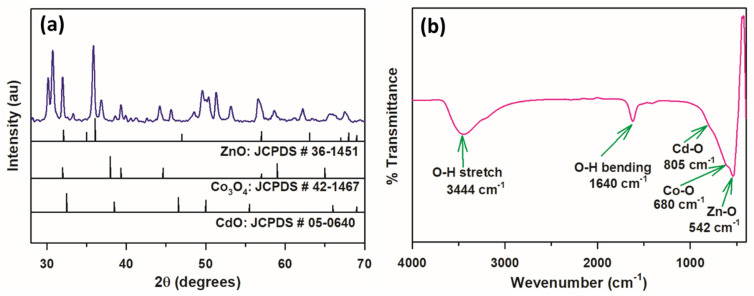
(**a**) XRD and (**b**) FTIR spectrum of CCZ nanoparticles.

The XRD pattern of the CCZ nanoparticles is displayed in Figure 1a. This pattern confirms the presence of the ZnO cubic phase. The diffraction peaks appearing at 2*θ* values of 32.1°, 35.0°, 36.0°, 47.1°, 57.0°, 63.2°, 67.0°, 67.9°, and 69.0° can be correlated to the planes (100), (002), (101), (102), (110), (103), (200), (112), and (201) of the cubic ZnO (JCPDS # 36-1451) [29,30,31], while diffraction peaks that appeared at 2*θ* values of 32.1°, 37.9°, 39.1°, 44.8°, 57.0°, 59.1°, and 65.0° can be assigned to (111), (220), (311), (222), (400), (422), (511), and (440) planes of cubic Co_3_O_4_, respectively (JCPDS # 42-1467, 03-65-3103) [32,33]. Again, diffraction peaks that appeared at 2*θ* values of 32.5°, 38.6°, 55.4°, and 66.0° can be assigned to the (111), (200), (311), (220), (311), and (222) planes of cubic CdO, respectively (JCPDS # 05-0640, 75-0592) [34,35]. These XRD peaks can be assigned to the standard Co_3_O_4_·CdO·ZnO cubic crystal phase.

The CCZ nanoparticles were further studied by FTIR to find out their atomic vibrations, as shown in Figure 1b. The ZnO displays an absorption band at 542 cm^−1^ in accordance with the metal–oxygen vibrational mode of absorption, which matches values in the literature [29,30,31]. The band at 680 cm^−1^ is due to the Co–O stressing mode of vibration [32,33]. The band appearing at 805 cm^−1^ is due to the Cd–O bond [34]. Two absorption bands appeared at 3444 and 1640 cm^−1^ belonging to absorbed water molecules [35].

**Figure 2:**
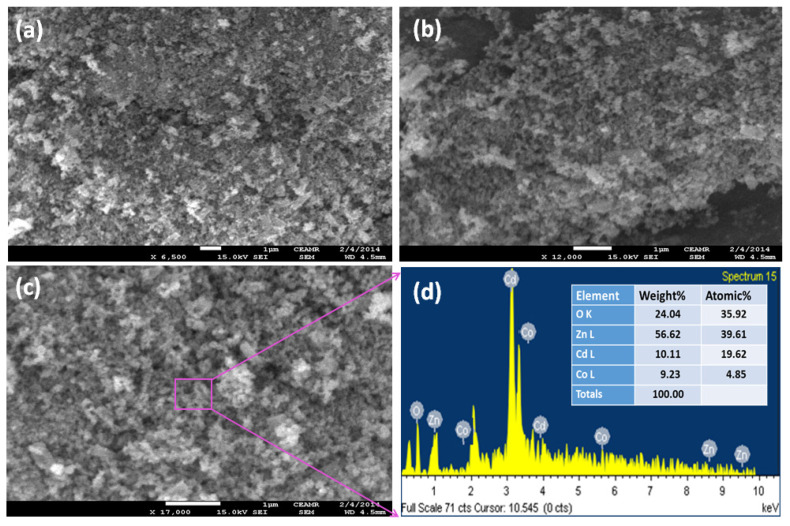
(**a**–**c**) Low- to high-resolution FE-SEM images and (**d**) EDS spectrum of the CCZ nanoparticles.

The morphological and surface structure of the CCZ nanoparticles was explored by FESEM (Figure 2a–c). The CCZ nanoparticles consist of Co_3_O_4_·CdO·ZnO, and aggregated a nano-particle-like morphological structure with micro-level size distributions. The FESEM image shows the nanoparticles of CCZ. The elemental composition of the CCZ nanoparticles was studied by EDS (Figure 2d), indicating that these CCZ nanoparticles consist of Zn, Co, Cd, and O, with respective weight percentages of 56.62%, 9.23%, 10.11%, and 24.04%.

**Figure 3:**
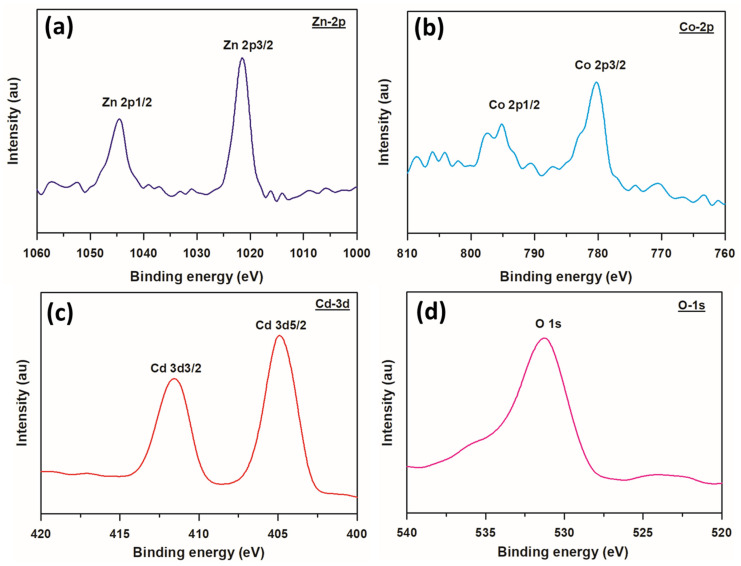
XPS study: Spectra of (**a**) Zn-2p, (**b**) Co-2p, (**c**) Cd-3d, and (**d**) O-1s orbital.

XPS was further used to obtain more information about the chemical bonds present in the CCZ nanoparticles. Figure 3a–d show the XPS spectra of Zn-2p, Co-2p, Cd-3d, and O-1s. In the Zn-2p spectrum (Figure 3a) peaks that appeared at 1021.5 and 1044.4 eV can be assigned to Zn2p3/2 and Zn2p1/2, respectively [30,31]. Thus, the XPS results establish that the active valency of Zn at the surface of ZnO is +2 [30]. This also shows that the Zn2p3/2 peak has a larger area, is narrower, and has a stronger band behavior than Zn2p1/2, which is in line with the standard ZnO. It is also noticed that the binding energy difference between the doublet peaks for Zn2p1/2 and Zn2p3/2 is ~23 eV for ZnO [36]. The Co-2p spectrum (Figure 3b) shows three peaks, with those that appeared at 780.2 and 795.3 eV related to Co2p3/2 and Co2p1/2, respectively [32,37,38]. The 14.58 eV energy difference between these peaks confirms the existence of both Co^2+^ and Co^3+^ states in Co_3_O_4_ [32,39]. The two distinct peaks (Figure 3c) at 404.8 and 411.5 eV are attributed to the Cd3d5/2 and Cd3d3/2 orbital, respectively [34,35]. The peak at 404.8 eV clearly indicates that Cd atoms are attached to oxygen atoms [40]. One asymmetric peak at 531.2 eV was obtained for the O-1s spectrum (Figure 3d) [41,42,43]. This asymmetry comes from the low and high binding energy components of different types of oxygen atoms [44]. Most of the fine scan XPS peaks are in line with the previous ZnO, Co_3_O_4_, and CdO reports. Thus, these XPS results further confirm the coexistence of Co_3_O_4_, CdO, and ZnO in the CCZ nanoparticles, which are well-matched with the EDS, FESEM, and TEM data.

**Figure 4:**
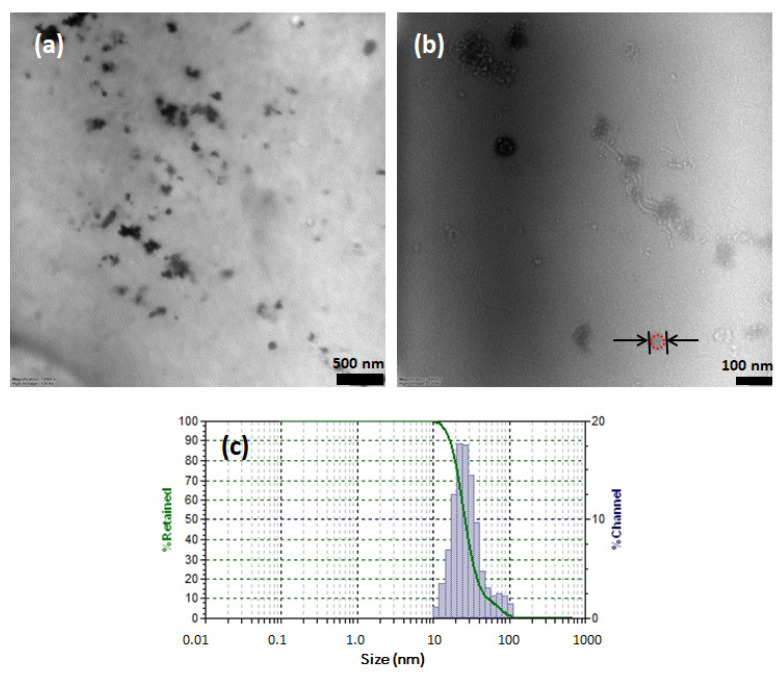
TEM and DLS investigation of CCZ nanoparticles. (**a**,**b**) Low- to high-magnified image and (**c**) DLS study.

The morphology of the CCZ nanoparticles was also evaluated using Tunneling Electron Microscopy (TEM), as presented in Figure 4. An aggregated spherical-shaped particle-like morphology is found via TEM analysis. The average diameter of the CCZ nanoparticles is 29.6 nm, in the range of 20.0 to 40.0 nm. The low and high magnified TEM images of the CCZ nanoparticles show the existence of aggregated nanoparticles (Figure 4a,b). Therefore, TEM images display the actual morphology of the CCZ nanoparticles in a spherical-shaped particle, which corresponds to the aggregation of ternary metal oxide nanoparticles. Additionally, we have also performed DLS on the prepared CCZ nanomaterials in the aqueous phase, and the result is presented in Figure 4c. The average size was found to be around 31.4 nm. It is observed that the size of the CCZ nanoparticles is a little larger in DLS than in the TEM analyses. DLS measures the hydrodynamic radius, which takes into account the interaction of the vesicle with water [45,46,47]. The CCZ particle size we get is the size of an equivalent sphere that diffuses similarly to a CCZ vesicle. However, TEM analysis (dried sample) gives the exact geometric radius, i.e., the radius without the hydration layer.

### 2.2. Methanol Sensor Development

#### 2.2.1. Detection of Methanol Using the CCZ/Au/μ-Chip Assembly

Toxic MeOH in the aqueous solution was detected by the CCZ-modified gold-coated μ-Chip as an electrochemical sensor. During electrochemical measurements, the MeOH produced a significant response when the MeOH touched the CCZ/Au/μ-Chip. Therefore, we have proposed a MeOH sensor based on the CCZ/Au/μ-Chip assembly in phosphate buffer solution (PBS). This will be the first MeOH sensor based on a CCZ/Au/μ-Chip assembly.

**Scheme 1:**
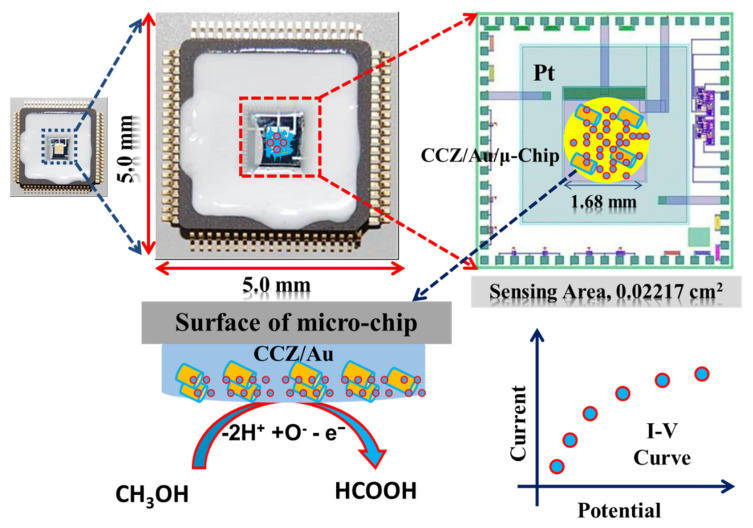
Proposed electrochemical oxidation mechanism of MeOH at the CCZ/Au/μ-Chip assembly.

In electrochemical detection, the current response was increased remarkably with the increasing MeOH concentration. During the electrochemical oxidation of MeOH n the CCZ/Au/μ-Chip assembly, one electron was transferred to the conduction band of the CCZ/Au/μ-Chip assembly by MeOH [48], which caused increases in the current response, as shown in Scheme 1.

Herein, a gold-coated microchip was modified with CCZ nanoparticles using PEDOT:PSS, and used in detecting MeOH. Here, the active sensing material, made of CCZ nanoparticles, consists of n-type ZnO and CdO [22,24] and p-type Co_3_O_4_ semiconductors [23], and thus provides synergistic effects through different oxidative-reductive active sites facilitating MeOH’s adsorption onto the fabricated electrode surface. Therefore, it can accept electrons from MeOH molecules easily, which ultimately facilitates MeOH oxidation. The low dimensions of the CCZ nanoparticles also enhance the interactions between the CCZ nanoparticles and MeOH molecules. Thus, when the MeOH molecule comes into contact with the CCZ/Au/μ-Chip surface, it loses one electron to the conduction band of the CCZ nanoparticles, and thus oxidize to methanoic acid, as presented in [49,50,51]. The CCZ/Au/μ-Chip electrode provides improved I-V responses compared to the Au/μ-Chip, indicating that the CCZ nanoparticles amplify the current responses during MeOH sensing. The existence of electron–hole pairs in the CCZ has expedited a promising nano-environment in MeOH detection, which may be the main reason for this superior sensor performance. Hence, we have concluded that these CCZ nanoparticles are very active in MeOH oxidation (Equation (1)).

CH_3_OH (O^−^_ads_) → HCOOH + 2H^+^ + e^−^ (1)

On the other hand, this mechanism can be explained according to the equations (Equations (2)–(7)). Initially, dissolved oxygen is chemisorbed onto the surface of the CCZ/Au/μ-Chip when methanol is put into the electrochemical solution. During the chemisorption, the surrounding oxygen form the ionic species (O^2−^ and O^−^), which gain electrons from the conduction band of the CCZ material. Therefore, methanol is oxidized to HCOH and subsequently HCOOH, and then liberates electrons into the conduction bank of CCZ, thereby decreasing the resistance of the CCZ/Au/μ-Chip substrate in the presence of CH_3_OH.

O_2_ (Dissolved) ↔ O_2_ (Adsorbed) (2)

O_2_ (Adsorbed) + e^−^ ↔ O^2−^ (Adsorbed) (3)

O^2−^ (Adsorbed) + e^−^ ↔ 2O^−^ (Adsorbed) (4)

CH_3_OH (gas) + O^−^ (Adsorbed) → HCOH + H_2_O + e^−^ (5)

HCOH + O (Bulk) → HCOOH + O (Vacancies)(6)

CH_3_OH (Analyte) + O_2_^−^ (Adsorbed) → HCOOH + H_2_O + e^−^ (7)

**Figure 5:**
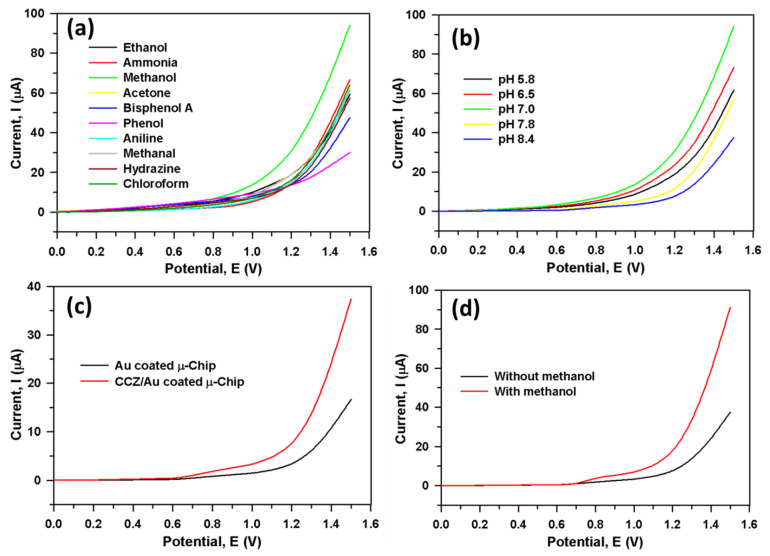
The current response of 10.0 µM; 25 µL using the CCZ/Au/μ-Chip electrode: (**a**) Selectivity study of ten interfering chemicals, (**b**) pH optimization, (**c**) Au/μ-Chip and CCZ/Au/μ-Chip electrode using 5.0 µM of MeOH, and (**d**) without and with the presence of MeOH.

Figure 5a displays the current responses for ten toxic interfering chemicals in the selectivity study, where aqueous MeOH (green line) in PBS gave a distinguishably higher current response in the CCZ/Au/μ-Chip assembly. Because of its ability to distinguish interfering agents from the MeOH with very similar electrochemical behaviors, the interference study is one of the most important methods in analytical chemistry, particularly for mixed metal oxide-based sensors. To study the effects of various interfering chemicals, the modified electrode was examined to check its acceptance in ambient conditions using 5.0 µM MeOH. From this, we can derive the highest concentrations of interfering substances that cause no more than 5% error. Thus, these electrochemical studies reveal that equal concentrations of ethanol, acetone, bisphenol A, phenol, aniline, hydrazine, and chloroform showed a negligible effect on the current response of MeOH. Therefore, it was confirmed that the CCZ/Au/μ-Chip assembly is selective towards MeOH in the presence of the above-mentioned interfering chemicals. The proposed CCZ/Au/μ-Chip sensor was appropriate for use in the determination of MeOH with high sensitivity. We have also studied the pH effect of the CCZ/Au/μ-Chip sensor in relation to MeOH for different pH values, ranging from 5.8 to 8.4 (Figure 5b). From the experiments, it is clear that the CCZ/Au/μ-Chip sensor displayed very good electrocatalytic activities at various pH values. Figure 5b, showing the pH effect with MeOH, reveals that at 7.0 pH (green line), the highest current output was observed. Therefore, pH ~7.0 was kept constant for the rest of the experiments of MeOH detection using the CCZ/Au/μ-Chip sensor. Figure 5c exhibits the current responses from 5.0 µM MeOH in PBS in a gold-coated μ-Chip (black line) and CCZ/Au/μ-Chip (red line). The CCZ/Au/μ-Chip electrode offered a considerably improved response compared to the Au/μ-Chip electrode, which confirms the exceptional electrochemical properties of the CCZ/Au/μ-Chip sensor in relation to MeOH under ambient conditions. Figure 5d displays the current output from the CCZ/Au/μ-Chip sensor with MeOH (red line) and in the absence of MeOH (black line). In the presence of MeOH, a substantial upsurge of output current was obtained, indicating the MeOH sensing ability of the CCZ/Au/μ-Chip sensor under ambient conditions.

**Figure 6:**
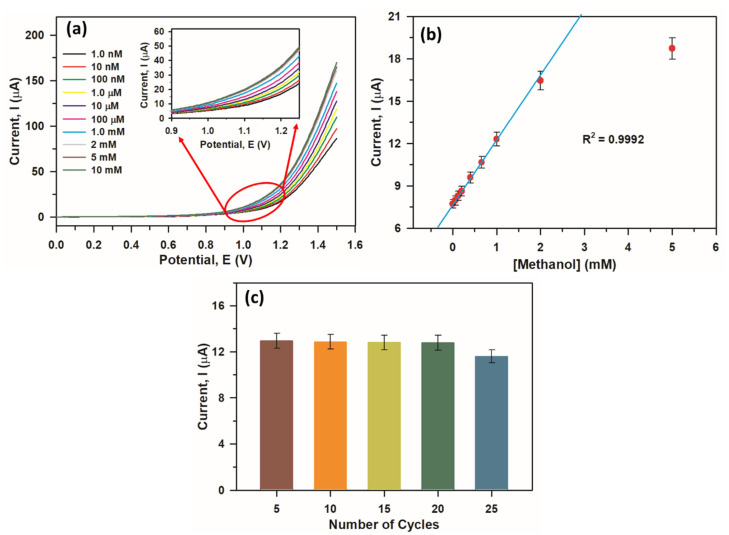
(**a**) Electrochemical responses for different MeOH solutions (1.0 nM to 10 mM), (**b**) calibration curve of the CCZ/Au/μ-Chip at +1.1 V, and (**c**) stability study.

We sequentially injected 25 µL of MeOH (1.0 nM to 10.0 mM) in 5.0 mL PBS; then, the variation in current response was investigated for each injection. Figure 6a shows the current responses from the CCZ/Au/μ-Chip for various MeOH concentrations ranging from 1.0 nM to 10.0 mM. It shows that the output current rises for the CCZ/Au/μ-Chip sensor with increasing MeOH concentrations. It is also seen that from a lower (1.0 nM) to a higher concentration (10.0 mM) of MeOH, the output current also rises gradually. A varying concentration of MeOH (1.0 nM to 10.0 mM) was used to select the linear dynamic range (LDR) and limit of detection (LOD) of the newly developed MeOH sensor. Figure 6b shows the calibration plot at +1.1 V, from which an extremely high sensitivity value was estimated as 1.3842 µAµM^−1^cm^−2^, while the LDR of the developed CCZ/Au/μ-Chip assembly was attained as 1.0 nM to 2.0 mM (R^2^ = 0.9992). An ultra-low LOD value was also obtained, as 32.8 ± 0.1 pM (3 × S/N).

Excellent reproducibility was achieved using five different CCZ/Au/μ-Chip electrodes under identical conditions, resulting in a relative standard deviation (RSD) of ~3.8%. The CCZ/Au/μ-Chip sensor’s repeatability was also tested for five successive runs in 5.0 μM MeOH, resulting in a current variance of RSD ~4.4%. After 30 days of electrode storage under room conditions, a nominal decrease in sensitivity and no physical damage were observed, all of which are useful in the practical use of this MeOH sensor. We have also checked the stability of the CCZ/Au/μ-Chip sensor for up to 25 cycles using 10.0 μM MeOH, as presented in Figure 6c. Stability investigations revealed that up to 20 cycles, the fabricated electrode retains excellent I-V response, but beyond that it gradually decreases.

#### 2.2.2. Investigation of Real Samples

For the functionality test, the CCZ/Au/μ-Chip sensor was used to detect MeOH in industrial ETP plant water (S1) and household wastewater (S2). We initially removed the solid particles from the real samples by filtration. Then, these real samples were analyzed using an electrochemical method, employing the newly developed CCZ/Au/μ-Chip sensor as the working electrode (WE). To this end, we employed the standard addition method in an aqueous medium to validate the correctness of the MeOH detection. Briefly, 25.0 µL of aqueous MeOH of various concentrations and an equal volume of real samples were mixed separately and studied in PBS by the CCZ/Au/μ-Chip sensor. Table 1 displays the results obtained, which show that the CCZ/Au/μ-Chip sensor achieved almost ~100% MeOH recovery. Therefore, we can conclude that this CCZ/Au/μ-Chip sensor is acceptable, accurate, and reliable in determining MeOH in real samples.

Electrochemical responses in MeOH detection depend primarily on the size, morphology, and surface structure of the electrode material. When the surface of the CCZ nanoparticles touched the MeOH molecules, a surface-mediated oxidation reaction occurred. Hence, MeOH molecules release electrons into the CCZ nanoparticles’ conduction band, which ultimately increases the CCZ/Au/μ-Chip sensor’s conductance [52,53,54,55]. Consequently, the electrochemical response increases. The CCZ/Au/μ-Chip sensor exhibited very high sensitivity in MeOH detection, and a significantly lower LOD than other MeOH sensors already published, as shown in Table 2. The CCZ/Au/μ-Chip sensor showed excellent stability and reliability as well.

The main features of the proposed CCZ/Au/μ-Chip sensor are high sensitivity, extremely wide LDR, long-term stability, an enhanced electrocatalytic property in detecting MeOH, a handy nature, good reproducibility, and low detection limit. Therefore, we can conclude that the CCZ/Au/μ-Chip sensor showed exceptional electron-facilitating behavior in MeOH detection.

## 3. Conclusions

We have successfully synthesized and characterized Co_3_O_4_·CdO·ZnO-based tri-metallic oxide nanoparticles (CCZ) using various conventional methods, including FTIR, XRD, TEM, DLS, FESEM, EDS, and XPS. Then, we developed an effective methanol sensor using a facile fabrication technique based on CCZ nanoparticle-fabricated gold-coated µ-Chips. The very high LDR and sensitivity value suggest that this CCZ/Au/µ-Chip-based methanol sensor can be used for the detection of a wide range of methanol concentrations. The ultra-low LOD value indicates that even a pico-molar level of methanol in environmental samples can be detected by this novel CCZ/Au/µ-Chip sensor. This CCZ/Au/µ-Chip sensor retains excellent electrochemical response up to 20 cycles, confirming its very good stability. Its other main features are short response time, excellent reproducibility, and repeatability. The standard addition method was used to validate the newly developed CCZ/Au/µ-Chip sensor with two environmental samples, including industrial effluent-water and waste-water, and obtained a reasonable quantitative ~100% recovery. Finally, a new route to the development of an efficient electrochemical sensor is introduced using tri-metallic oxide nanoparticle-embedded µ-Chip devices for the safety of the healthcare and environmental field.

## 4. Experimental

### 4.1. Materials and Methods

Hydrated zinc chloride, cobalt (II) chloride, cadmium (II) chloride, ammonium hydroxide, methanol, PEDOT:PSS, ethanol, acetone, bisphenol A, phenol, aniline, hydrazine, chloroform, etc., used in the current study were from Sigma-Aldrich (St. Louis, MO, USA), and all of them were used as received. For CCZ nanoparticles, the FTIR spectrum was studied by NICOLET iS50 FTIR spectrometer (Thermo Scientific, Waltham, MA, USA). The powder XRD prototypes of the CCZ nanoparticles were studied by an X-ray diffractometer (XRD, Thermo scientific, ARL X’TRA diffractometer). The XPS values of CCZ nanoparticles were recorded using an MgKα spectrometer (JEOL, JPS 9200) with an excitation radiation source (MgKα, pass energy = 50.0 eV, voltage = 10 kV, current = 20 mA). The morphology of the CCZ nanoparticles was studied by FESEM (JEOL, JSM-7600F, Japan), TEM (JEM 1400Flash, JEOL, Tokyo, Japan) and DLS (SZ-100, Horiba, Japan). The elemental analysis was performed using the EDS from JEOL, Japan. The I-V method was carried out by the Keithley 6517A Electrometer (Tx, USA) at normal temperature.

### 4.2. Synthesis of the CCZ Nanoparticles

We synthesized the CCZ nanoparticles by a simple solution method. Briefly, in this reaction, equimolar (0.1 M) Zn^2+^, Co^2+^, Cd^2+^ and NH_4_OH were used. These ions were mixed (50 mL each) in a 250 mL conical flask for half an hour with continuous stirring at 60 °C. Then, we added 100 mL of aqueous NH_4_OH (0.1 M) drop-wise to this mixture with constant stirring. We continued the stirring for 6 h at 70 °C. Upon cooling, a gray precipitate of CCZ nanoparticles was produced. This was later washed with double-distilled water and ethanol. Then, we dried this precipitate at ambient conditions for half an hour. After that, we heated the precipitate for 2 h at 65 °C to get the as-grown CCZ nanoparticles.

### 4.3. Fabrication of Au/μ-Chip with the CCZ Nanoparticles

In this approach, the modification of the Au/μ-Chip was performed via CCZ nanoparticles using sol-gel drop coating (Dispersed CCZ in Ethanol:Acetone mixture as 1:1 ratio), and then by PEDOT:PSS (10.0 μL). Then, we dried the fabricated Au/μ-Chip under ambient conditions for 1 h to obtain a thin film on the Au/μ-Chip. In an electrochemical cell, a fabricated CCZ/Au/μ-Chip, Pt-wire, and MeOH solution in PBS (pH 7.0) were used as the WE, counter electrode, and target analyte, respectively. MeOH solution (10.0 mM) was taken as a stock solution for the targeted analyte, and electrochemical methods were engaged to detect MeOH.

## Figures and Tables

**Table 1:** Investigation of environmental samples by the CCZ/Au/μ-Chip sensor using an electrochemical approach.

Sample	MeOH Conc. Added	MeOH Conc. Determined by CCZ/Au/µ-Chip	Recovery (%)	RSD (%) (*n* = 3)
S1	2.000 nM	2.058 nM	102.9	3.6
	2.000 µM	2.096 µM	104.8	4.2
S2	2.000 nM	1.976 nM	98.8	3.9
	2.000 µM	1.982 µM	99.1	4.4

**Table 2:** Comparison of different electrochemical sensors for MeOH detection.

Material	Method	LDR (mM)	LOD (mM)	Sensitivity (μAmM^−1^cm^−2^)	Ref.
α-Fe_2_O_3_- CdSe	I-V	0.2–800	0.041	0.2744	[48]
Pt-ZnO NSp	Amp	30–1000	30	0.235	[56]
PMMA-G-CNTs	I-V	1.0 *–10	0.39 *	13.491	[57]
Pd–Ni/SiNWs	Amp	0–75	0.025	480	[58]
PTh/α-Fe_2_O_3_	I-V	5–100	1.59	0.793	[59]
CCZ/Au/μ-Chip	I-V	1.0 *–2.0	32.8 **	1.3842 ***	This work

* = nM, ** = pM, *** = μAμM^−1^cm^−2^, NSp = nanospheres, G = graphene, SiNWs = silicon nanowires, PTh = poly thiophine, PMMA = polymethyl methacrylate, Amp = amperometry.

## Data Availability

Data will be available upon request.

## References

[1] Qiu Q., Jiang N., Ge L., Li X., Chen X. (2020). The Electrochemical Sensor for Methanol Detection Based on Trimetallic PtAuAg Nanotubes. J. Mater. Sci..

[2] Sivakumar C., Berchmans S. (2018). Methanol Electro-Oxidation by Nanostructured Pt/Cu Bimetallic on Poly 3,4 Ethylenedioxythiophene (PEDOT). Electrochim. Acta.

[3] Xu H., Yan B., Zhang K., Wang C., Zhong J., Li S., Yang P., Du Y. (2017). Facile Synthesis of Pd-Ru-P Ternary Nanoparticle Networks with Enhanced Electrocatalytic Performance for Methanol Oxidation. Int. J. Hydrog. Energy.

[4] Yoshizawa T., Kamijo Y., Fujita Y., Suzuki Y., Hanazawa T., Usui K., Kishino T. (2018). Mild Manifestation of Methanol Poisoning Half a Day after Massive Ingestion of a Fuel Alcohol Product Containing 70% Ethanol and 30% Methanol: A Case Report. Acute Med. Surg..

[5] Chan A.P.L., Chan T.Y.K. (2018). Methanol as an Unlisted Ingredient in Supposedly Alcohol-Based Hand Rub Can Pose Serious Health Risk. Int. J. Environ. Res. Public Health.

[6] Ahumada L.A.C., Cruz A.F.S., Fox M.A.G., Sandoval O.L.H. (2018). Useful Piezoelectric Sensor to Detect False Liquor in Samples with Different Degrees of Adulteration. J. Sensors.

[7] Akbari E., Buntat Z., Nikoukar A., Kheirandish A., Khaledian M., Afroozeh A. (2016). Sensor Application in Direct Methanol Fuel Cells (DMFCs). Renew. Sustain. Energy Rev..

[8] Zhang X., Chen Y. (2009). A Light-Modulated Chemosensor for Methanol with Ratiometry and Colorimetry. Anal. Chim. Acta.

[9] Wrobel K., Rodríguez D.M., Aguilar F.J.A., Wrobel K. (2005). Determination of Methanol in o,o-Dimethyldithiophosphoric Acid (DMDTPA) of Technical Grade by UV/Vis Spectrophotometry and by HPLC. Talanta.

[10] Hoyos-Arbeláez J., Vázquez M., Contreras-Calderón J. (2017). Electrochemical Methods as a Tool for Determining the Antioxidant Capacity of Food and Beverages: A Review. Food Chem..

[11] Maduraiveeran G., Sasidharan M., Ganesan V. (2018). Electrochemical Sensor and Biosensor Platforms Based on Advanced Nanomaterials for Biological and Biomedical Applications. Biosens. Bioelectron..

[12] Manikandan V.S., Adhikari B.R., Chen A. (2018). Nanomaterial Based Electrochemical Sensors for the Safety and Quality Control of Food and Beverages. Analyst.

[13] Greeley J., Mavrikakis M. (2004). Competitive Paths for Methanol Decomposition on Pt(111). J. Am. Chem. Soc..

[14] Du X., Luo S., Du H., Tang M., Huang X., Shen P.K. (2016). Monodisperse and Self-Assembled Pt-Cu Nanoparticles as an Efficient Electrocatalyst for the Methanol Oxidation Reaction. J. Mater. Chem. A.

[15] Wen D., Guo S., Wang Y., Dong S. (2010). Bifunctional Nanocatalyst of Bimetallic Nanoparticle/TiO2 with Enhanced Performance in Electrochemical and Photoelectrochemical Applications. Langmuir.

[16] Naresh N., Karthik P., Vinoth R., Muthamizhchelvan C., Neppolian B. (2018). Tailoring Multi-Metallic Nanotubes by Copper Nanowires with Platinum and Gold via Galvanic Replacement Route for the Efficient Methanol Oxidation Reaction. Electrochim. Acta.

[17] Safavi A., Maleki N., Moradlou O. (2008). A Selective and Sensitive Method for Simultaneous Determination of Traces of Paracetamol and P-Aminophenol in Pharmaceuticals Using Carbon Ionic Liquid Electrode. Electroanalysis.

[18] Huang W., Hu W., Song J. (2003). Adsorptive Stripping Voltammetric Determination of 4-Aminophenol at a Single-Wall Carbon Nanotubes Film Coated Electrode. Talanta.

[19] Maalej N.M., Qurashi A., Assadi A.A., Maalej R., Shaikh M.N., Ilyas M., Gondal M.A. (2015). Synthesis of Gd_2_O_3_:Eu Nanoplatelets for MRI and Fluorescence Imaging. Nanoscale Res. Lett..

[20] Singh S.K., Kumar K., Rai S.B. (2009). Multifunctional Er^3+^-Yb^3+^ Codoped Gd_2_O_3_ Nanocrystalline Phosphor Synthesized through Optimized Combustion Route. Appl. Phys. B Lasers Opt..

[21] Hirai T., Orikoshi T. (2004). Preparation of Gd_2_O_3_:Yb,Er and Gd_2_O_2_S:Yb,Er Infrared-to-Visible Conversion Phosphor Ultrafine Particles Using an Emulsion Liquid Membrane System. J. Colloid Interface Sci..

[22] Ahmed J., Faisal M., Jalalah M., Alsareii S.A., Harraz F.A. (2021). Novel Polypyrrole-Carbon Black Doped ZnO Nanocomposite for Ef Fi Cient Amperometric Detection of Hydroquinone. J. Electroanal. Chem..

[23] Vladimirova S., Krivetskiy V., Rumyantseva M., Gaskov A., Mordvinova N., Lebedev O., Martyshov M., Forsh P. (2017). Co_3_O_4_ as P-Type Material for CO Sensing in Humid Air. Sensors.

[24] Anitha M., Deva Arun Kumar K., Mele P., Anitha N., Saravanakumar K., Sayed M.A., Ali A.M., Amalraj L. (2021). Synthesis and Properties of p-Si/n-Cd1−xAgxO Heterostructure for Transparent Photodiode Devices. Coatings.

[25] Adeosun W.A., Asiri A.M., Marwani H.M., Rahman M.M. (2020). Enzymeless Electrocatalytic Detection of Uric Acid Using Polydopamine/Polypyrrole Copolymeric Film. ChemistrySelect.

[26] Sheikh T.A., Rahman M.M., Asiri A.M., Marwani H.M. (2018). Sensitive 3-Chlorophenol Sensor Development Based on Facile Er_2_O_3_/CuO Nanomaterials for Environmental Safety. New J. Chem..

[27] Rahman M.M., Alam M.M., Asiri A.M. (2018). Selective Hydrazine Sensor Fabrication with Facile Low-Dimensional Fe_2_O_3_/CeO_2_ Nanocubes. New J. Chem..

[28] Rahman M.M., Khan S.B., Faisal M., Asiri A.M., Tariq M.A. (2012). Detection of Aprepitant Drug Based on Low-Dimensional Un-Doped Iron Oxide Nanoparticles Prepared by a Solution Method. Electrochim. Acta.

[29] Ahmed J., Rahman M.M., Siddiquey I.A., Asiri A.M., Hasnat M.A. (2017). Efficient Bisphenol-A Detection Based on the Ternary Metal Oxide (TMO) Composite by Electrochemical Approaches. Electrochim. Acta.

[30] Ahmed J., Rahman M.M., Siddiquey I.A., Asiri A.M., Hasnat M.A. (2018). Efficient Hydroquinone Sensor Based on Zinc, Strontium and Nickel Based Ternary Metal Oxide (TMO) Composites by Differential Pulse Voltammetry. Sens. Actuators B Chem..

[31] Rahman M.M., Ahmed J., Asiri A.M., Alamry K.A. (2020). Fabrication of a Hydrazine Chemical Sensor Based on Facile Synthesis of Doped NZO Nanostructure Materials. New J. Chem..

[32] Abu-Zied B.M., Alam M.M., Asiri A.M., Ahmed J., Rahman M.M. (2020). Efficient Hydroquinone Sensor Development Based on Co_3_O_4_ Nanoparticle. Microchem. J..

[33] Aboelazm E.A., Gomaa A.M., Ali K.F.C. (2018). Cobalt Oxide Supercapacitor Electrode Recovered from Spent Lithium-Ion Battery. Chem. Adv. Mater..

[34] Kaviyarasu K., Manikandan E., Paulraj P., Mohamed S.B., Kennedy J. (2014). One Dimensional Well-Aligned CdO Nanocrystal by Solvothermal Method. J. Alloys Compd..

[35] Karthik K., Dhanuskodi S., Gobinath C., Prabukumar S., Sivaramakrishnan S. (2019). Multifunctional Properties of CdO Nanostructures Synthesised through Microwave Assisted Hydrothermal Method. Mater. Res. Innov..

[36] Al-Gaashani R., Radiman S., Daud A.R., Tabet N., Al-Douri Y. (2013). XPS and Optical Studies of Different Morphologies of ZnO Nanostructures Prepared by Microwave Methods. Ceram. Int..

[37] Rahman M.M., Ahmed J. (2018). Cd-Doped Sb_2_O_4_ Nanostructures Modified Glassy Carbon Electrode for Efficient Detection of Melamine by Electrochemical Approach. Biosens. Bioelectron..

[38] Rahman M.M., Ahmed J., Asiri A.M. (2018). Thiourea Sensor Development Based on Hydrothermally Prepared CMO Nanoparticles for Environmental Safety. Biosens. Bioelectron..

[39] Abu-Zied B.M. (2019). A Novel Foam Combustion Approach for the Synthesis of Nano-Crystalline Cobalt Oxide Powder. Ceram. Int..

[40] Liu L., Jiao Y., Gao C., Xu H., Zhao W., Dai W., Yu W., Li X. (2018). Improving the Performance of Cu_2_Zn(Sn_y_Ge_1-y_)(S_x_Se_1-x_)4 Solar Cells by CdS:Zn Buffer Layers. J. Alloys Compd..

[41] Ahmed J., Faisal M., Harraz F.A., Jalalah M., Alsareii S.A. (2021). Porous Silicon-Mesoporous Carbon Nanocomposite Based Electrochemical Sensor for Sensitive and Selective Detection of Ascorbic Acid in Real Samples. J. Taiwan Inst. Chem. Eng..

[42] Ahmed J., Rashed A., Faisal M., Harraz F.A., Jalalah M., Alsareii A. (2021). Applied Surface Science Novel SWCNTs-Mesoporous Silicon Nanocomposite as Efficient Non-Enzymatic Glucose Biosensor. Appl. Surf. Sci..

[43] Ahmed J., Faisal M., Jalalah M., Alsaiari M., Alsareii S.A., Harraz F.A. (2021). An Efficient Amperometric Catechol Sensor Based on Novel Polypyrrole-Carbon Black Doped α-Fe_2_O_3_ Nanocomposite. Colloids Surf. A Physicochem. Eng. Asp..

[44] Ren H., Xiang G., Gu G., Zhang X. (2014). Enhancement of Ferromagnetism of ZnO:Co Nanocrystals by Post-Annealing Treatment: The Role of Oxygen Interstitials and Zinc Vacancies. Mater. Lett..

[45] Khan A., Parwaz Khan A.A., Rahman M.M., Asiri A.M., Alamry K.A. (2015). Preparation of Polyaniline Grafted Graphene Oxide–WO3 Nanocomposite and Its Application as a Chromium(Iii) Chemi-Sensor. RSC Adv..

[46] Arshad M.N., Sheikh T.A., Rahman M.M., Asiri A.M., Marwani H.M., Awual M.R. (2017). Fabrication of Cadmium Ionic Sensor Based on (E)-4-Methyl-N′-(1-(Pyridin-2-Yl)Ethylidene)Benzenesulfonohydrazide (MPEBSH) by Electrochemical Approach. J. Organomet. Chem..

[47] Rahman M.M., Abu-Zied B.M., Hasan M.M., Asiri A.M., Hasnat M.A. (2016). Fabrication of a Selective 4-Amino Phenol Sensor Based on H-ZSM-5 Zeolites Deposited Silver Electrodes. RSC Adv..

[48] Abdullah M.M., Faisal M., Ahmed J., Harraz F.A., Jalalah M., Alsareii S.A. (2021). Sensitive Detection of Aqueous Methanol by Electrochemical Route Using Mesoporous α-Fe_2_O_3_ Doped CdSe Nanostructures Modified Glassy Carbon Electrode. J. Electrochem. Soc..

[49] Crosnier de Lassichere C., Latapie L., Evrard D., Gros P. (2018). New Insight into the EC’ Mechanism of Uric Acid Regeneration in the Presence of Ascorbic Acid on a Poly(3,4-Ethylenedioxithiophene) Modified Gold Electrode. Electroanalysis.

[50] Khan M.M.I., Haque A.M.J., Kim K. (2013). Electrochemical Determination of Uric Acid in the Presence of Ascorbic Acid on Electrochemically Reduced Graphene Oxide Modified Electrode. J. Electroanal. Chem..

[51] Pessoa J., Sárkány Z., Ferreira-da-Silva F., Martins S., Almeida M.R., Li J., Damas A.M. (2010). Functional Characterization of Arabidopsis Thaliana Transthyretin-like Protein. BMC Plant Biol..

[52] Rahman M.M., Khan S.B., Jamal A., Faisal M., Asiri A.M. (2012). Fabrication of a Methanol Chemical Sensor Based on Hydrothermally Prepared α-Fe_2_O_3_ Codoped SnO_2_ Nanocubes. Talanta.

[53] Faisal M., Khan S.B., Rahman M.M., Jamal A., Abdullah M.M. (2012). Fabrication of ZnO Nanoparticles Based Sensitive Methanol Sensor and Efficient Photocatalyst. Appl. Surf. Sci..

[54] Rahman M.M., Khan S.B., Jamal A., Faisal M., Asiri A.M. (2012). Highly Sensitive Methanol Chemical Sensor Based on Undoped Silver Oxide Nanoparticles Prepared by a Solution Method. Microchim. Acta.

[55] Rahman M.M., Khan S.B., Asiri A.M. (2014). Smart Methanol Sensor Based on Silver Oxide-Doped Zinc Oxide Nanoparticles Deposited on Microchips. Microchim. Acta.

[56] Ahmad M., Gan L., Pan C., Zhu J. (2010). Controlled Synthesis and Methanol Sensing Capabilities of Pt-Incorporated ZnO Nanospheres. Electrochim. Acta.

[57] Rahman M.M., Hussein M.A., Alamry K.A., Al Shehry F.M., Asiri A.M. (2016). Sensitive Methanol Sensor Based on PMMA-G-CNTs Nanocomposites Deposited onto Glassy Carbon Electrodes. Talanta.

[58] Tao B., Zhang J., Hui S., Chen X., Wan L. (2010). An Electrochemical Methanol Sensor Based on a Pd-Ni/SiNWs Catalytic Electrode. Electrochim. Acta.

[59] Harraz F.A., Faisal M., Jalalah M., Almadiy A.A., Al-Sayari S.A., Al-Assiri M.S. (2020). Conducting Polythiophene/α-Fe_2_O_3_ Nanocomposite for Efficient Methanol Electrochemical Sensor. Appl. Surf. Sci..

